# Supplementary material for: Brain Connectivity Modelling Through Joint Estimation of Parcels and Gradients
Source: bioRxiv. 2026 Jun 28:2026.06.23.734045. Preprint. [Version 1] doi: 10.64898/2026.06.23.734045 (PMC13320991; doi:10.64898/2026.06.23.734045)
Supplement: Supplement 1 [file NIHPP2026.06.23.734045v1-supplement-1.pdf]

bioRxiv preprint doi: https://doi.org/10.64898/2026.06.23.734045; this version posted June 28, 2026. The copyright holder for this preprint (which was not certified by peer review) is the author/funder, who has granted bioRxiv a license to display the preprint in perpetuity. It is made available under a CC-BY 4.0 International license.



# A Appendix

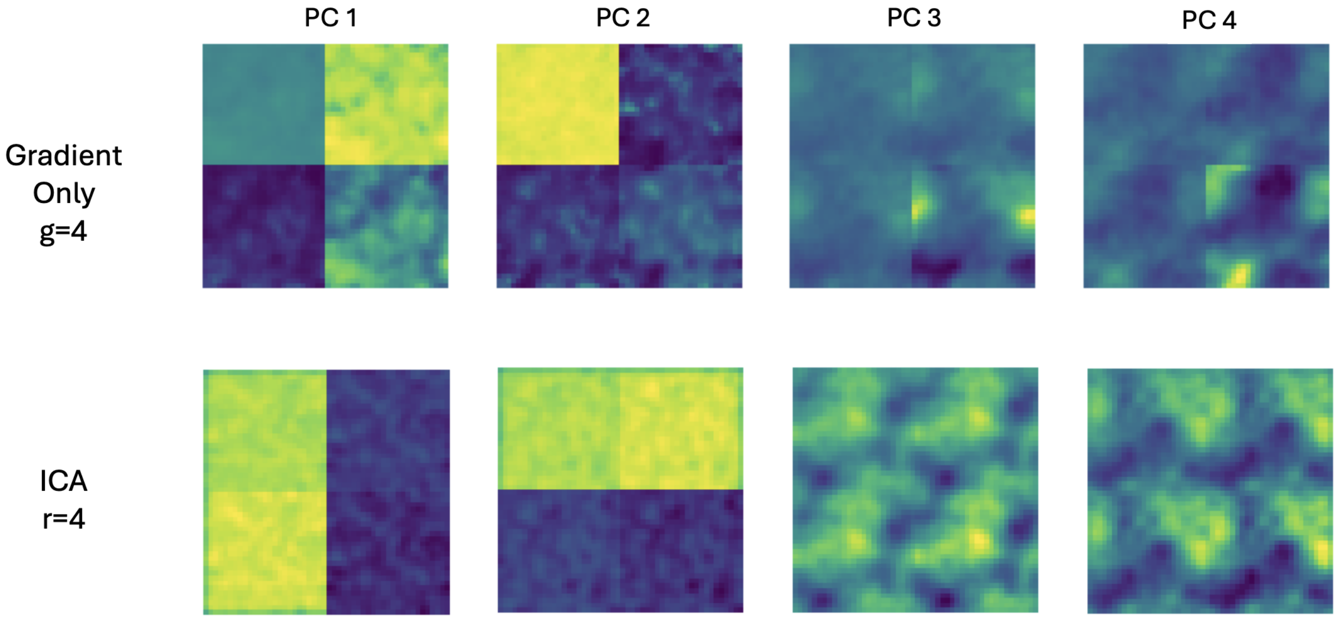


Figure S1: The top 4 principal gradients [15], as well as the spatial ICA maps for synthetic data.

bioRxiv preprint doi: https://doi.org/10.64898/2026.06.23.734045; this version posted June 28, 2026. The copyright holder for this preprint (which was not certified by peer review) is the author/funder, who has granted bioRxiv a license to display the preprint in perpetuity. It is made available under a CC-BY 4.0 International license.



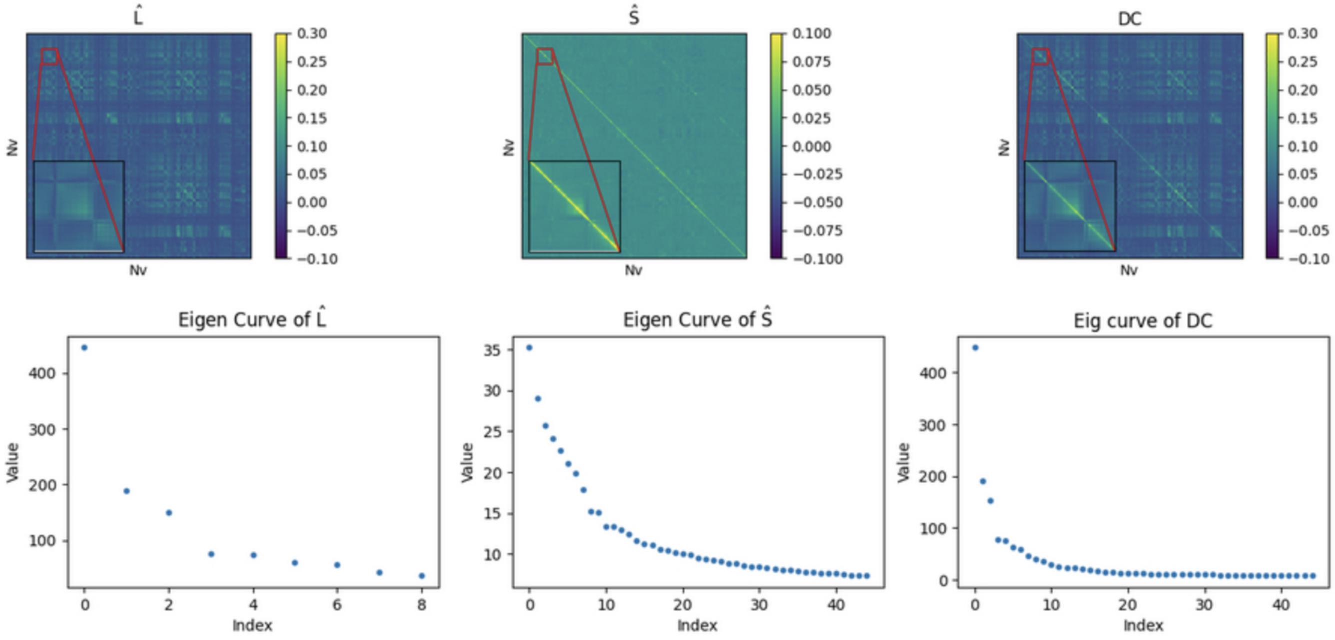


Figure S2: Estimated $\mathbf{L}$ and $\mathbf{S}$ matrices from HCP dataset, visualised together along with the empirical DC and their eigen-curves.

bioRxiv preprint doi: https://doi.org/10.64898/2026.06.23.734045; this version posted June 28, 2026. The copyright holder for this preprint (which was not certified by peer review) is the author/funder, who has granted bioRxiv a license to display the preprint in perpetuity. It is made available under a CC-BY 4.0 International license.



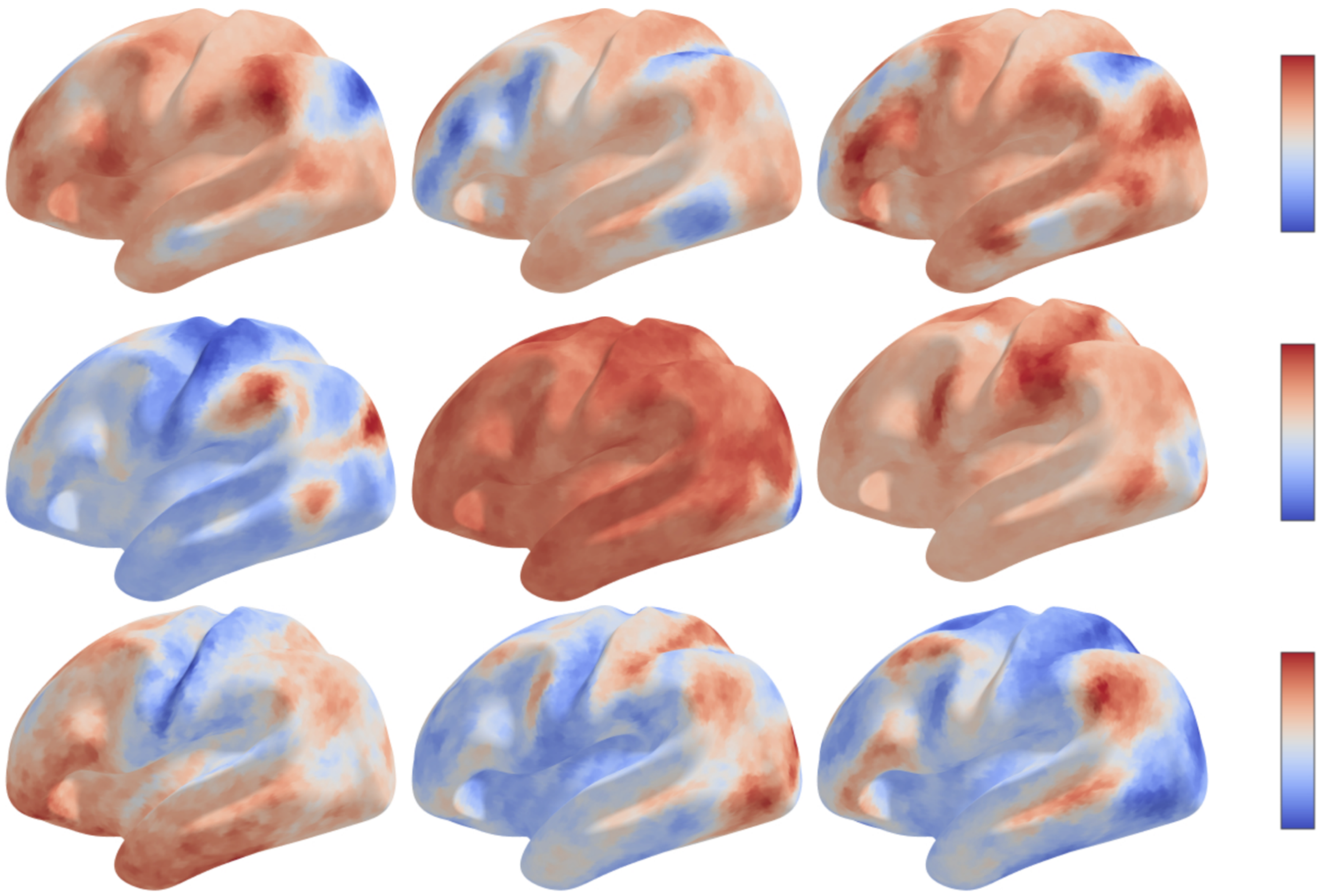

Figure S3: ICA components of $\hat{\mathbf{L}}$ ($r_m = 9$) for HCP dataset.

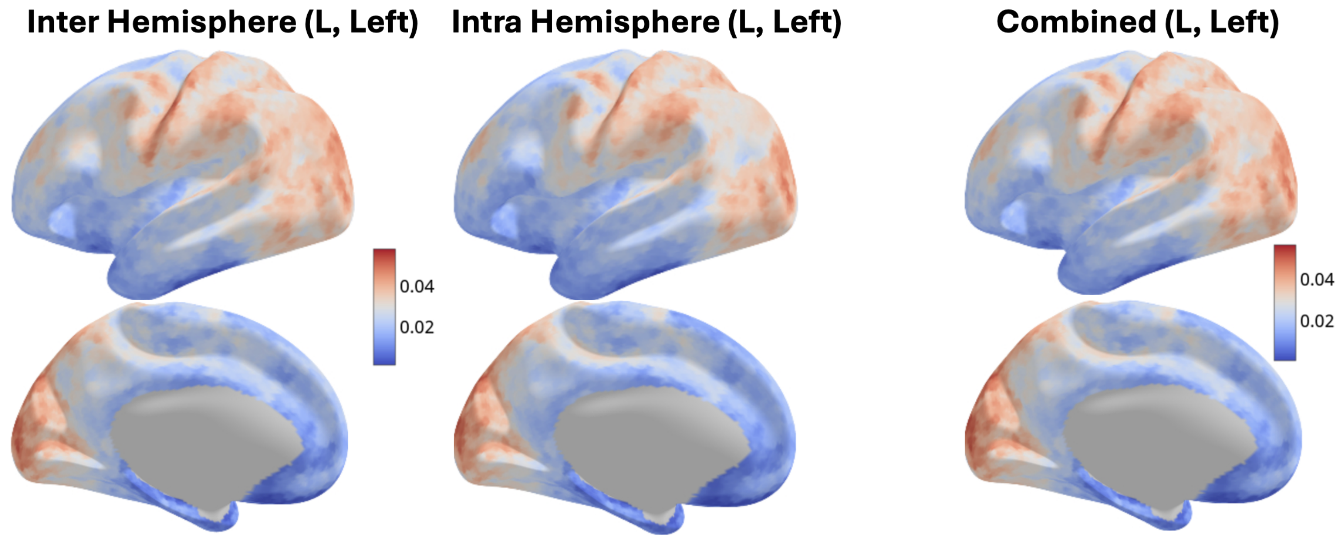


Figure S4: The average inter- vs intra-hemispheric vs combined connectivity maps in $\hat{\mathbf{L}}$ visualised for left hemisphere (HCP dataset).

bioRxiv preprint doi: https://doi.org/10.64898/2026.06.23.734045; this version posted June 28, 2026. The copyright holder for this preprint (which was not certified by peer review) is the author/funder, who has granted bioRxiv a license to display the preprint in perpetuity. It is made available under a CC-BY 4.0 International license.



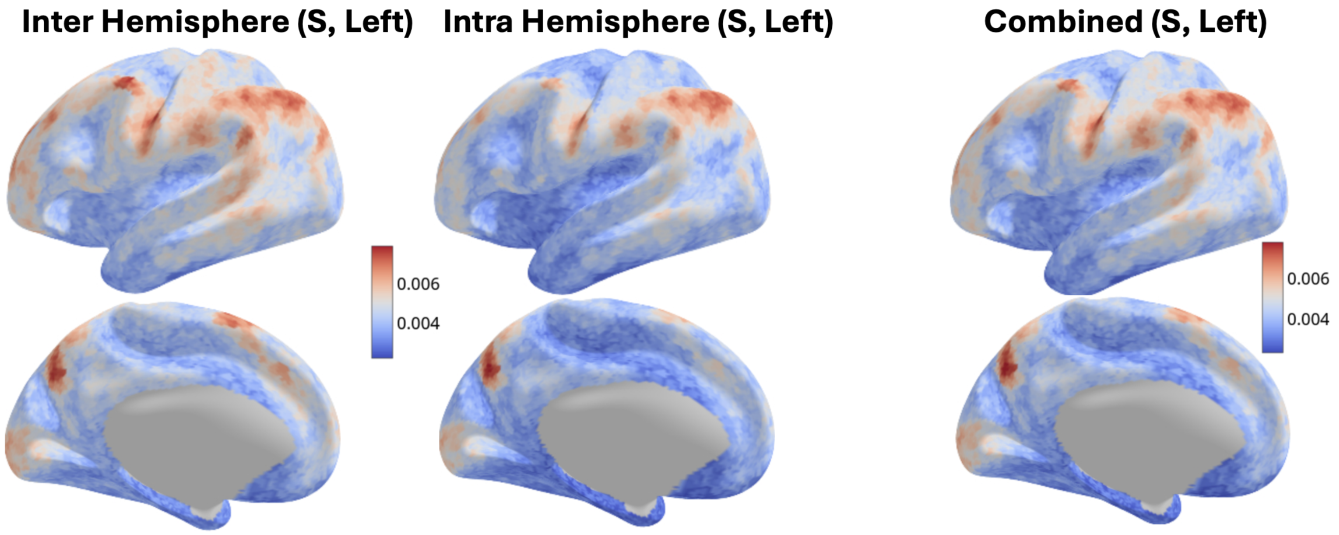


Figure S5: The average inter- vs intra-hemispheric vs combined connectivity maps in $\hat{\mathrm{S}}$ visualised for left hemisphere (HCP dataset).
